# Investigation of Factors Influencing Infant Mortality at Greater Accra Regional Hospital, Ghana

**DOI:** 10.1155/2024/6610617

**Published:** 2024-04-08

**Authors:** Gabriel Kallah-Dagadu, Foster Donkor, Magdalene Duah, Hillary Yeboah, Dennis Arku, Anani Lotsi

**Affiliations:** Department of Statistics and Actuarial Science, University of Ghana, Accra, Ghana

## Abstract

**Background:**

Annually, 5.4 million children under five face mortality, with 2.5 million deaths in the first month, 1.6 million between one and eleven months, and 1.3 million aged one to four. Despite global strides, sub-Saharan Africa, including Ghana, grapples with persistent high child mortality. This study employs statistical methods to pinpoint factors driving under-five mortality in the Greater Accra Regional Hospital.

**Methods:**

The data was acquired from Greater Accra Regional Hospital, Ghana, spanning January to December 2020. The data comprised all under-five deaths recorded in the hospital in 2020. The statistical tools employed were the chi-square test of association and the multinomial logistic regression model.

**Results:**

In 2020, there were 238 cases of under-five mortality recorded in the hospital, with males constituting the majority (55%). About 85% of these cases occurred within the first month of birth, primarily attributed to respiratory distress, prematurity, and sepsis. Notably, meconium aspiration was the least common among grouped diagnoses. The test of association and multinomial logistic model emphasised the child's age, birth type, and weight at birth as significant factors influencing child mortality. Conversely, attributes like sex, marital status, and mother's age displayed no notable association with the diagnosis of death.

**Conclusion:**

The study on child mortality at the Greater Accra Regional Hospital unveils key factors shaping child health outcomes, emphasising the role of age, birth type, and weight. While specific demographics show no significant association, identified predictors are vital for targeted interventions. Proposed strategies encompass education programs, improved care, birthing practices, and data-driven policies.

## 1. Introduction

Despite commendable progress achieved under the millennium development goals, sub-Saharan Africa grapples with persistently high under-five mortality rates. The UNICEF 2022 report highlights a global under-five mortality rate (U5MR) of 38 deaths per 1,000 live births in 2021, yet in sub-Saharan Africa, this figure soars to 74 deaths per 1,000 live births [1]. In the specific case of Ghana, the U5MR stands at 44 per 1,000 live births, nearly doubling the Sustainable Development Goal (SDG) target set for 2030 [1]. The United Nations Inter-Agency Group for Child Mortality Estimation (2023) revealed that as of 2021, the probability per 100 that a newborn baby will die before reaching age five is 4.5% for Ghana and lower-middle-income countries and little above 7% for sub-Saharan Africa. However, this indicator's Sustainable Development Goal target is 2.5 deaths per 100 live births [2].

Child mortality, especially in those under five, remains a pressing global and public health issue [3], enduring alongside the challenges posed by the COVID-19 pandemic in 2020. Despite considerable strides in the past two decades in reducing mortality among children and early adolescents, an estimated 6.3 million children and young adolescents succumbed to largely preventable causes in 2019 [4]. Of these fatalities, 5.4 million were children under the age of five, with 2.5 million occurring in the first month of life, emphasising the vulnerability of neonates. Although 118 countries had already achieved under-five mortality rates below the SDG target in 2017, approximately 50 countries, including many in sub-Saharan Africa, face the challenge of accelerating progress to meet the SDG target by 2030 [5]. The Global Burden of Disease Collaborative Network estimated the annual number of deaths for children under five from each cause in Ghana. The top causes of death are malaria, neonatal asphyxia and trauma, neonatal sepsis and infections, lower respiratory infections, congenital disabilities, neonatal preterm birth, and diarrheal diseases [6].

Ghana, while exhibiting a declining trend in under-five mortality since the 1970s, grapples with insufficient progress despite the implementation of national health policies such as the Healthcare Policy 2007-2018, community-based health planning and service, and Universal Health Coverage [7, 8]. Despite this, Ghana fell short of meeting the fourth Millennium Development Goal (MDG4) [9]. The rate of under-five mortality, although decreasing, remains a significant concern, especially in rural areas where rates surpass those in urban areas. Regional disparities within Ghana are evident, with under-five mortality ranging from 47 per 1,000 live births in Greater Accra to a staggering 111 per 1,000 live births in the Northern Region. The highest rates are concentrated in Northern Ghana, followed by the Upper West and Ashanti regions [10]. A retrospective study conducted at the Komfo Anokye Teaching Hospital in Ghana recorded very high neonatal in-patient mortality. Out of 5,195 neonatal admissions recorded between 2013 and 2014, neonatal mortality rate was 20.3% (1,053) [11].

This situation becomes particularly distressing because preventive measures could have averted many of these deaths. Consequently, understanding the root causes of under-five mortality becomes imperative for informing healthcare policy and preventive measures aligned with the UN Sustainable Development Goals. Despite health surveys indicating a decline in under-five mortality, there is a critical need for comprehensive information on the specific factors contributing to this decrease, particularly in the study focusing on data from the Greater Accra Regional Hospital. This study employs statistical methods to pinpoint factors driving under-five mortality in the Greater Accra Regional Hospital of Ghana.

## 2. Materials and Methods

This study retrospectively designed the collection of hospital records over one year. We collected the hospital records of children aged zero to less than five years who died in the Greater Accra Regional Hospital in 2020 from their hospital folders. We considered all children's cases that occurred in the Greater Accra Regional Hospital, and the primary outcome variable was under-five child mortality. The hospital health biostatisticians in the children's department retrieved the data from the children's folders. After receiving a written proposal of the study and an introductory letter from the head of the Department of Statistics and Actuarial Science, the hospital management approved the data release. We chose this hospital because it serves as a referral hospital for the Greater Accra Region, catering to a broad community and providing access to all tribes of Ghana. Additionally, the hospital's location in the central part of Accra, the capital city of Ghana, influenced the selection.

The study considered several variables that affect under-five mortalities, and these include the following: maternal characteristics, child characteristics, delivery care, and diagnosis of death. The outcome variable was the diagnosis of the death of the child. The mother's characteristics included age at delivery, marital status, place of residence, and national health insurance status. The children's characteristics were age in weeks, weight at birth, sex, and birth type.

We used Excel and R statistical software to clean and prepare the data. We analysed the descriptive statistics using frequency distribution tables and bar graphs. We employed the chi-square test for association to test the relationship between the dependent variable (diagnosis of death) and the child and mother's characteristics. We categorised the diagnosis of death into six levels and used the identified significant predictors from the chi-square test to build a multinomial logistic regression model. We would use the Wald test to assess the significant contributions of the predictors in the multinomial logistic regression model. We would use odds ratios (OR) to measure each predictor's contribution to each diagnosis of death, the response variable. Finally, we will perform multicollinearity diagnostics to check for collinearity among the predictor variables using variance inflation factor (VIF) and tolerance statistics based on the SPSS version 29.

## 3. Results and Discussion

### 3.1. Results

In this section, we present the study's findings through descriptive statistics, using bar graphs and frequency distribution tables to represent the distribution of critical variables visually. We employed statistical techniques such as the chi-square test of association and the multinomial logistic regression model to achieve the study's objectives.

We performed several preprocessing steps on the data to improve the clarity of analysis. We categorised mothers' ages into three groups: young (15-24 years), adult (25-40 years), and older adult (above 40 years). Similarly, we segmented the child's age into four categories, one week (0-7 days), two weeks (8-14 days), four weeks (15-28 days), and beyond one month, considering a significant number of deaths occurring before the child reached one month. We also classified child weight into three levels: underweight (<2.5 kg), normal (2.5-4.0 kg), and overweight (>4.0 kg). We grouped the diagnosis of death into six categories over the study period, birth asphyxia (34; 14.3%), meconium aspiration (18; 7.6%), prematurity (43; 18.1%), respiratory distress (84; 35.3%), sepsis (41; 17.2%), and others (18; 7.6%), which included less frequent diagnoses. In total, we recorded 238 deaths during the study period.

**Figure 1:**
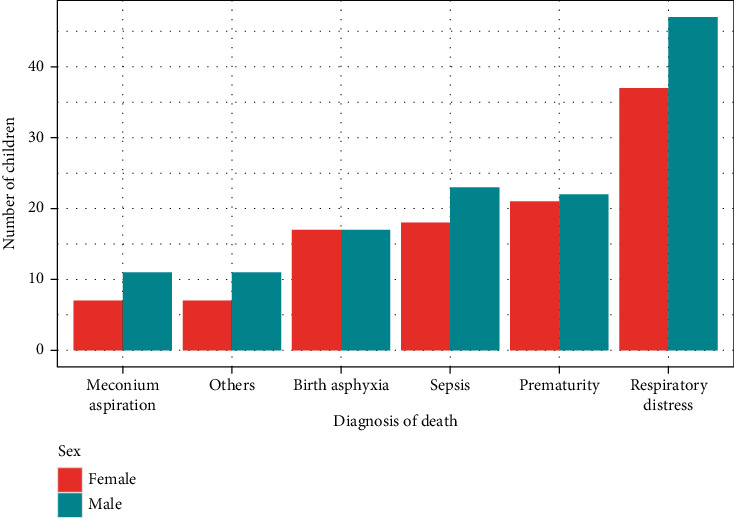
Distribution of diagnosis of death against the number of children.

Figure 1 illustrates the graphical distribution of diagnosis of death against the number of children by sex. The bar graph reveals that respiratory distress is the leading cause of death, predominantly affecting males. Prematurity and sepsis emerged as the second most common diagnosis of death among children at the Greater Accra Regional Hospital in 2020. Among the grouped diagnoses, meconium aspiration stands out as the least common cause of death.

**Figure 2:**
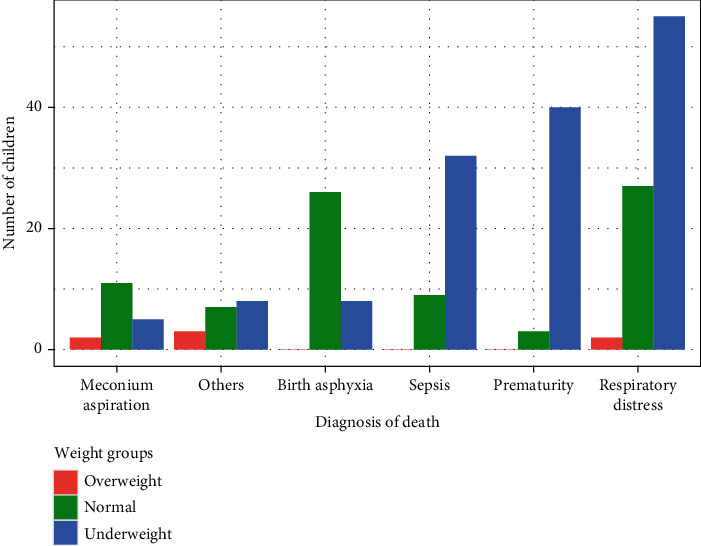
Distribution of diagnosis of death by the weight of the children at birth.

**Figure 3:**
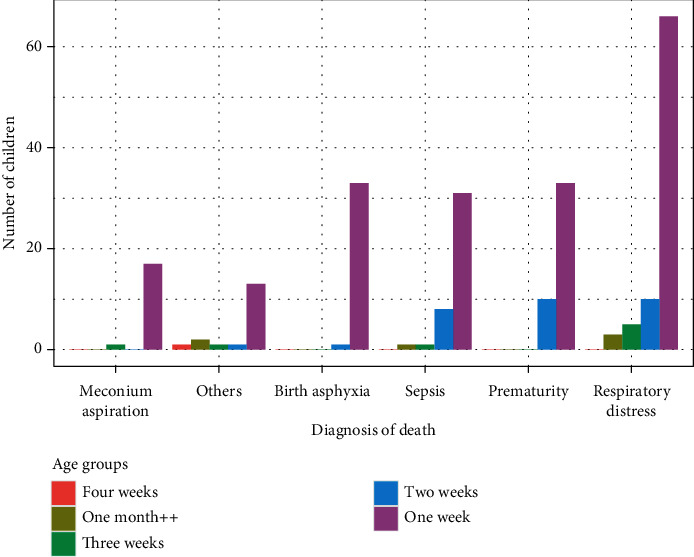
Distribution of diagnosis of death by the age of the children in weeks.

Furthermore, Figure 2 delves into the association between low birth weight (underweight) and specific diagnosis of death. It reveals that children born with low weight are most susceptible to respiratory distress, prematurity, and sepsis as diagnosis of death.  Figure 3 explores the distribution of diagnosis of death based on the age grouping of children in weeks. A noteworthy observation is that approximately 85 percent of deaths occurred before children reached four weeks, emphasising the critical vulnerability during this early period. The results underscore that all diagnoses of death considered in this study were notably prevalent in the first week of children born at the study hospital, indicating a concentration of health risks during this initial timeframe.

Table 1 presents a comprehensive examination of the association between the cause of a child's death and various demographic characteristics of both the child and the mother. Notably, the analysis revealed that sex, marital status, and the mother's age exhibited no statistically significant association with the cause of the child's death. However, crucial associations were identified concerning the child's age, birth type, and birth weight, all demonstrating statistical significance with *p* values less than 0.05.

The significance of these associations underscores that the age of the child, the method of birth, and the birth weight are influential factors affecting under-five child mortality. These variables, identified as statistically significant predictors, carry substantial weight in understanding and predicting child mortality outcomes. Recognising the impact of these factors is vital for healthcare practitioners, policymakers, and researchers, as it provides valuable insights into the nuanced interplay between demographic characteristics and diagnosis of child mortality. This nuanced understanding can contribute to developing targeted interventions and policies to improve child health outcomes and reduce mortality rates.

Table 2 provides a comprehensive overview of the multinomial logistic regression model, examining the relationship between the cause of a child's death and key predictors, including age, birth type, and weight. Birth asphyxia serves as the reference category for the cause of death. The results underscore the statistical significance of the predictors, specifically with prematurity (involving birth type and age), respiratory distress (involving birth type and weight), sepsis (involving all three predictors), and others (also involving all three predictors) at a 0.05 significance level. The analysis reveals the substantial impact of these predictors on the likelihood of different diagnoses of death, providing valuable insights into the complex interplay of factors influencing child mortality.  Table 3 displays a multicollinearity analysis of the predictors and shows that no multicollinearity among the independent variables exists. The VIF statistics for the three predictors were all less or equal to 1.00, and the tolerance statistics were also greater than 0.9, implying no multicollinearity.

Of note, birth type exhibits notably large odds ratios (OR) to respiratory distress, sepsis, and others, underscoring its significant role in shaping the outcomes of these diagnoses of death. This finding emphasises the importance of considering birth type as a critical factor in understanding and predicting specific diagnoses of child mortality. By elucidating these associations, the multinomial logistic regression model contributes to a subtlety understanding of the intricate dynamics surrounding child mortality, offering essential information for healthcare practitioners and policymakers to tailor interventions effectively.

### 3.2. Discussion

Despite significant progress in Ghana's healthcare, services, and infrastructure development, the country continues to face persistently high child mortality rates [12, 13]. The study utilised statistical methods to investigate the factors contributing to under-five mortality at the Greater Accra Regional Hospital. The overarching aim was to provide insights that could inform healthcare policies and preventive measures aligned with the UN Sustainable Development Goals, specifically targeting the reduction of under-five mortality rates.

Table 1's assessment of the association between demographic characteristics and diagnosis of death reveals that sex, marital status, and mother's age exhibit no significant association with the cause of a child's death, disagreeing with most studies conducted on a national scale [14]. However, sex, which was not significant in the diagnosis of death, agrees with Adam's [15] study conducted at 37 Military Hospital Ghana. Furthermore, the age of the child, birth type, and weight at birth were associated with the diagnosis of death, agreeing with other studies conducted in teaching hospitals in Ghana [11, 16]. These findings suggest that understanding a child's age, birth circumstances, and weight is pivotal for comprehending and predicting the causes of mortality. These significant predictors should be integral to healthcare strategies and policies to reduce child mortality rates.

The study's findings underscored acute respiratory distress as a prominent cause of death among under-five children, followed by premature births and sepsis, which is in agreement with the global burden of diseases [6] and other hospital-based studies [11, 15, 16]. Supporting these findings, a systematic review and meta-analysis conducted by Wong et al. [17] revealed a 24% overall mortality rate in pediatrics related to acute respiratory distress syndrome (ARDS). Wong et al. [17] further demonstrated an improving trend in the mortality rates of pediatric ARDS, highlighting the potential underestimation of past incidence due to diagnostic challenges. Lehtonen et al. [18] argued that most causes of child mortality, including congenital disabilities, sudden infant death syndrome, premature births, and birth asphyxia, are often beyond the control of healthcare professionals. Notably, neonatal sepsis emerged as a global concern, particularly in sub-Saharan Africa, where it accounts for 26% of under-five deaths, as highlighted in a study by Liu et al. [19]. Additionally, research by Adatara et al. [20] conducted at Trauma and Specialist Hospital in Winneba, Ghana, indicated a heightened probability of neonatal sepsis with increasing neonatal age.

The chi-square test of association highlighted a significant link between the age, birth type, and weight of children and the diagnosis of child mortality according to several studies in sub-Saharan Africa [21–23]. According to WHO [24] data, significant contributors to under-five deaths include preterm birth complications, pneumonia, birth asphyxia, diarrhea, and malaria. Malnutrition, accounting for 45% of child deaths, is particularly pronounced in sub-Saharan Africa, where child mortality rates are over 14 times higher than in developed regions. Neonatal sepsis stands out as a significant factor, constituting 38% of global neonatal deaths in the region [24].

Moreover, the multinomial logistic regression model in Table 2 extends the analysis, affirming the significance of predictors like birth type, age, and weight in influencing specific death diagnoses. Notably, the model indicates substantial odds ratios for birth type concerning respiratory distress, sepsis, and others, highlighting its critical role in shaping outcomes for these diagnoses of death. This model provided more profound insights into specific risk factors contributing to child mortality. For meconium aspiration, the child's age emerged as a more significant factor than weight and birth type. Negative coefficients for multiple births and child weight in meconium aspiration indicated their adverse impact compared to birth asphyxia.

Conversely, positive coefficients for the child's age and weight in prematurity suggested a higher likelihood of these factors causing premature death. We identified that the child's age, weight, and multiple births are more likely diagnoses of respiratory distress than birth asphyxia. Additionally, multiple births and the child's age were positively associated with other diseases, leading to under-five child mortality. In contrast, the child's weight showed a negative association compared to birth asphyxia.

While the study provides valuable insights into child mortality at the Greater Accra Regional Hospital, it is essential to acknowledge its potential limitations. One notable limitation is the study's retrospective nature, relying on historical data, which introduces the possibility of incomplete or inaccurate records, impacting the reliability of the findings. In addition, the generalizability of the study findings may also be limited since the data was specific to the Greater Accra Regional Hospital, and the scope of the study was limited to only 2020, which may introduce some potential bias. The healthcare infrastructure, socioeconomic factors, and cultural influences in the Greater Accra region might differ significantly from those in other areas, as argued by Marmot and Bell [25] and Aheto [26], affecting the study's external validity.

Furthermore, the study's reliance on hospital records may miss child mortality cases outside healthcare facilities resulting in fewer cases above one month after delivery. Not capturing all deaths within the hospital setting can lead to an underestimation of the true prevalence of under-five child mortality in the community. Another potential limitation is that we did not explore contextual factors such as socioeconomic status, maternal education, household wealth, and access to healthcare services, which previous research has identified as risk factors for under-five mortality [14, 21, 23]. These factors play a crucial role in child health outcomes but may have needed to have been adequately addressed in the study, limiting the depth of the analysis.

The study's findings suggest several targeted interventions and future strategies to address child mortality at the Greater Accra Regional Hospital. Firstly, implementing health education programs for mothers and caregivers, explicitly focusing on critical predictors like the child's age, birth type, and weight at birth, could empower parents with essential knowledge for infant care [21]. Strengthening prenatal and postnatal care services, especially for high-risk groups, and improving birthing practices through healthcare professional training can enhance the overall quality of care provided during childbirth. Additionally, community outreach and awareness campaigns can extend healthcare initiatives to communities, promoting preventive measures and early recognition of danger signs. Policymakers are encouraged to use the study's data to inform evidence-based policy development, allocating resources effectively to address the identified predictors of child mortality.

Continued research efforts are vital to understanding contextual factors influencing child mortality. Exploring socioeconomic determinants, maternal education, and community-level factors can provide a more comprehensive understanding of the complex dynamics contributing to child mortality in the Greater Accra Region. By implementing these multifaceted strategies and considering the identified predictors, healthcare stakeholders can work collaboratively towards reducing child mortality rates and improving the overall health outcomes for children in the region.

## 4. Conclusion

In conclusion, the study on child mortality at the Greater Accra Regional Hospital provides valuable insights into the multifaceted factors influencing child health outcomes. The findings highlight the significance of predictors such as the child's age, birth type, and weight at birth. While specific demographic characteristics like sex, marital status, and mother's age showed no significant association with the diagnosis of a child's death, the identified predictors serve as crucial considerations for targeted interventions.

Possible interventions and future strategies include implementing health education programs, strengthening prenatal and postnatal care, improving birthing practices, conducting community outreach initiatives, and using data-driven policies to address the region's unique dynamics of child mortality. The study underscores the need for a comprehensive approach considering specific predictors and tailored interventions to the local context. We also emphasise the importance of continued research into contextual factors to enhance our understanding and inform evidence-based strategies for reducing child mortality rates in the Greater Accra Region. Overall, the study provides a foundation for informed decision-making, offering actionable insights for healthcare practitioners, policymakers, and researchers working towards improving child health outcomes.

## Figures and Tables

**Table 1:** Chi-square test of association between the diagnosis of death and demographic characteristics of child and mother.

Variables	Birth asphyxia	Meconium aspiration	Prematurity	Respiratory distress	Sepsis	Others	Total	*p* value
*Sex*								0.946
Female	17 (50.0%)	7 (38.9%)	21 (48.8%)	37 (44.0%)	18 (43.9%)	7 (38.9%)	107 (45.0%)	
Male	17 (50.0%)	11 (61.1%)	22 (51.2%)	47 (56.0%)	23 (56.1%)	11 (61.1%)	131 (55.0%)	
*Age in weeks*								0.023
One week	33 (97.1%)	17 (94.4%)	33 (76.7%)	66 (78.6%)	31 (75.6%)	13 (72.2%)	193 (81.1%)	
Two weeks	1 (2.9%)	0 (0.0%)	10 (23.3%)	10 (11.9%)	8 (19.5%)	1 (5.6%)	30 (12.6%)	
Four weeks	0 (0.0%)	1 (5.6%)	0 (0.0%)	5 (6.0%)	1 (2.4%)	2 (11.1%)	9 (3.8%)	
Above four weeks	0 (0.0%)	0 (0.0%)	0 (0.0%)	3 (3.6%)	1 (2.4%)	2 (11.1%)	6 (2.5%)	
*Birth type*								0.024
Single	34 (100.0%)	18 (100.0%)	37 (86.0%)	70 (83.3%)	34 (82.9%)	18 (100.0%)	211 (88.7%)	
Multiple (twins)	0 (0.0%)	0 (0.0%)	6 (14.0%)	14 (16.7%)	7 (17.1%)	0 (0.0%)	27 (11.3%)	
*Weight at birth*								<0.05
Normal (2.5-4.0 kg)	26 (76.5%)	13 (72.2%)	3 (7.0%)	27 (32.1%)	9 (22.0%)	8 (44.4%)	86 (36.1%)	
Overweight (>4.0 kg)	0 (0.0%)	0 (0.0%)	0 (0.0%)	2 (2.4%)	0 (0.0%)	2 (11.1%)	4 (1.7%)	
Underweight (≤2.5 kg)	8 (23.5%)	5 (27.8%)	40 (93.0%)	55 (65.5%)	32 (78.0%)	8 (44.4%)	148 (62.2%)	
*Marital status*								0.672
Married	29 (85.3%)	13 (72.2%)	35 (81.4%)	64 (76.2%)	29 (70.7%)	13 (72.2%)	183 (76.9%)	
Single	5 (14.7%)	5 (27.8%)	8 (18.6%)	20 (23.8%)	12 (29.3%)	5 (27.8%)	55 (23.1%)	
*Mother's age*								0.161
15-24 years	2 (5.9%)	3 (16.7%)	5 (11.6%)	10 (11.9%)	8 (19.5%)	4 (22.2%)	32 (13.4%)	
25-40 years	31 (91.2%)	14 (77.8%)	35 (81.4%)	68 (81.0%)	32 (78.0%)	10 (55.6%)	190 (79.8%)	
Above 40 years	1 (2.9%)	1 (5.6%)	3 (7.0%)	6 (7.1%)	1 (2.4%)	4 (22.2%)	16 (6.7%)	

**Table 2:** Coefficient estimates of the multinomial logistic regression model.

Diagnosis of death	Predictors	Estimate (*ß*)	Std. error	OR	Wald stat	*p* value
Meconium aspiration	Intercept	-0.61	0.89		-0.69	0.50
	Birth type	-3.78	0.01	0.02	-378.00	<0.05
	Age	0.02	0.09	1.02	0.22	0.81
	Weight	-0.03	0.29	0.97	-0.10	0.91

Prematurity	Intercept	-1.44	0.87		-1.66	0.11
	Birth type	-4.57	0.01	0.01	-457.00	<0.05
	Age	0.17	0.07	1.18	2.43	0.02
	Weight	0.05	0.29	1.05	0.17	0.87

Respiratory distress	Intercept	2.62	0.67		3.91	<0.05
	Birth type	13.23	0.34	5.50*E*+05	38.91	<0.05
	Age	0.11	0.07	1.12	1.57	0.12
	Weight	-1.5	0.29	0.23	-5.17	<0.05

Sepsis	Intercept	1.79	0.62		2.89	<0.05
	Birth type	13.73	0.29	9.20*E*+05	47.34	<0.05
	Age	0.14	0.07	1.16	2.00	0.04
	Weight	-0.66	0.22	0.52	-3.00	<0.05

Others	Intercept	1.95	0.67		2.91	<0.05
	Birth type	13.52	0.33	7.50*E*+05	40.97	<0.05
	Age	0.14	0.07	1.15	2.00	0.04
	Weight	-1.14	0.27		-4.22	<0.05

**Table 3:** Model analysis for multicollinearity diagnostics and statistics.

Model collinearity	Dimension	Eigenvalue	Condition index	Variance proportions			
				Constant	Age	Weight	Birth type
Diagnostics	1	3.12	1.00	0.00	0.03	0.02	<0.05
	2	0.72	2.09	0.00	0.96	0.02	<0.05
	3	0.15	4.50	0.03	0.01	0.96	0.03
	4	0.01	15.10	0.96	0.00	0.01	0.97

Statistics	Tolerance	—	—	—	1.00	0.95	0.95
	VIF	—	—	—	1.00	1.06	1.06

## Data Availability

Permission to use the data was obtained from the management of Greater Accra Regional Hospital, Ghana. The data is available upon request and has no personal identifiers.
